# Supplementary material for: Interleukin-35 impairs human NK cell effector functions and induces their ILC1-like conversion with tissue residency features
Source: Nat Commun. 2025 Jul 3;16:6135. doi: 10.1038/s41467-025-61196-0 (PMC12229632; doi:10.1038/s41467-025-61196-0)
Supplement: Supplementary file 1 — Supplementary Information [file 41467_2025_61196_MOESM1_ESM.pdf]

SUPPLEMENTAL DATA

Supplemental Table 1: Flow cytometry antibodies

Antibodies	Clone	Reference	Supplier	Dilution
**Surface**				
**CD3-BV711**	UCHT1	563725	BD Biosciences	1/40
**CD3-PECy7**	UCHT1	563423	BD Biosciences	1/40
**CD9-Pe-Cy7**	HI9a	312116	Biolegend	1/35
**CD14-BV711**	M5E2	301838	Biolegend	1/40
**CD16-FITC**	3G8	555406	BD Biosciences	1/50
**CD19-BV711**	HIB19	302246	Biolegend	1/40
**CD25-BB515**	2A3	564467	BD Biosciences	1/45
**CD49a-APC**	TS2/7	328314	Biolegend	1/30
**CD56-APC**	NCAM16.2	341027	BD Biosciences	1/40
**CD56-VioBright-FITC**	AF12-7H3	130-113-309	Miltenyi	1/40
**CD69-APCFire750**	FN50	310946	Biolegend	1/50
**CD96-PE**	6F9	562379	BD Biosciences	1/30
**CD103-BV421**	Ber-ACT8	350214	Biolegend	1/30
**CD103-PE**	Ber-ACT8	350206	Biolegend	1/30
**CD127 (IL-7Ra)-APC-Cy7**	A019D5	351348	Biolegend	1/30
**CD130-PECF594**	AM64	564152	BD Biosciences	1/40
**CD137 (41BB)-PE**	4B4-1	309804	Biolegend	1/20
**CD159a (NKG2A)-APC**	REA110	130-113-563	Miltenyi	1/40
**CD159c (NKG2C)-BUV737**	134591	749685	BD Biosciences	1/20
**CD223(LAG3)-PECF594**	T47-530	565718	BD Biosciences	1/35
**CD226 (DNAM-1)-BV421**	11A8	338331	Biolegend	1/40
**CD274 (PD-L1)-BV711**	29e.2A3	329722	Biolegend	1/30
**CD314 (NKG2D)-BV650**	1D11	563408	BD Biosciences	1/35
**CD335 (NKp46)-BV510**	9E2	564064	BD Biosciences	1/30
**CD650 (NKp46)-BV650**	9E2	331927	Biolegend	1/30
**CD336 (NKp44)-PE-Cy7**	p44-8	325116	Biolegend	1/30
**CD337 (NKp30)-BV711**	p30-15	563383	BD Biosciences	1/30
**CD366 (TIM-3)-BV421**	7D3	565562	BD Biosciences	1/30
**IL12RB2-PE**	S16020B	394206	Biolegend	1/40
**IL27RA-APC**	W16093B	384608	Biolegend	1/40
**TIGIT-PE-Cy7**	A15153G	372714	Biolegend	1/30
**Intracellular**				
**IFN-γ-BV421**	4S-B3	502532	Biolegend	1/45
**IFN-γ-PE**	B27	554701	BD Biosciences	1/45
**Transcription factor**				
**T-bet-PECF 594**	O4-46	562467	BD Biosciences	1/20
**Eomes-PE**	X4-83	566749	BD Biosciences	1/40

Supplemental Table 2: Summary of analysis parameters used for quality control, standard pre-processing and downstream analysis for each of the scRNAseq datasets integrated into the pan-cancer atlas.

($n.UMI$ = threshold of raw counts ; $n.\ genes$ = threshold of genes ; $percent.\ mt$ = threshold of feature proportions associated with the mitochondrial genome ; $n.\ cells$ = number of cells remaining after filtering ; $n.\ features$ = number of variable features identified for dimensional reduction ; $n.\ dims$ = number of principal components retained for UMAP embeddings ; $k.\ param$ = nearest neighbor parameter value for SNN graph construction ; res = granularity resolution parameter for Louvain cluster identification)

	QC Metrics Thresholds			Standard Preprocessing Settings		Downstream Analysis Parameters		
**Dataset ID**	n.UMI	n.gene	percent.mt	n.cells	n.features	n.dims	k.param	res
GSE131907	500	200	20%	48160	2000	25	20	0.8
GSE160269	500	500	20%	190517	2000	30	30	0.9
GSE166555	1000	500	10%	36298	2000	20	20	0.8
GSE176078	250	200	20%	79404	3000	35	10	1.5

Supplemental figure legends

Supplementary Fig. 1 | IL-35 is a potent, dose-dependent, modulator of human NK cells

a-b) MFI expression of IFN-γ and CD25 in the presence of IL-35 relative to control without IL-35 for each cytokine activation condition. Mean values ± S.D are shown (n = 4 individual donors). **c)** Frequency of viable NK cells in the presence of IL-35 relative to control without IL-35 for each cytokine activation condition. Mean values ± S.D are shown (n = 4 individual donors). **d-e)** NK cells were isolated from the blood of healthy donors and activated for 24 h with IL-12+IL-18, alone or in combination with increasing doses of IL-35 (0 to 500 ng/mL) prior to analysis of intracellular IFN-γ and CD25 expression by flow cytometry. Representative flow cytometry plots (left) and quantification (%) (right) of IFN-γ intracellular expression and CD25 surface expression in NK cells 24h after cytokine activation. Mean values ± S.D are shown (n = 5 individual donor). **f)** Representative heat mapping of surface marker intensity for IFN-γ, CD25, 4-1BB, LAG3, PD-L1, CD69 expression in NK cells after 24h of culture in the presence of Medium and IL-12+IL-18, with or without IL-35. **g)** Raw data of cytokines and chemokines levels quantified by ECLIA in the supernatants from NK cells that were cultured for 24h in medium, IL-12+IL-18 and IL-15+IL-18 with or without IL-35. Mean values ± S.D are shown (n = 4 to 6 individual donors). **h)** Raw data of T-BET and EOMES expression analyzed by flow cytometry in NK cells that were cultured for 24h in medium, IL-12+IL-18 and IL-15+IL-18 with or without IL-35. Mean values ± S.D are shown (n = 6 or 7 individual donors). Source data are provided as a Source Data file.

Supplementary Fig. 2 | IL-35 inhibits NK cell IL-2-induced proliferation

a) Representative flow cytometry plots for CD25 and CD56 expression (left) and quantification of % CD25-expressing NK cells (right) after 16h-pre-activation in medium, IL-12, IL-15, IL-12+IL-15. Mean values ± S.D are shown (n=7 experiments). **b)** Representative illustrative images of culture wells of NK cells, pre-activated for 16h with indicated treatment, and then cultured in IL-2 with (red) or without (green) IL-35 for 3, 5, 7, and 9 days (D3, D5, D7 and D9 respectively). Source data are provided as a Source Data file.

Supplementary Fig. 3 | Long-term exposure to IL-35 leads to NK cells hyporesponsiveness and regulates the expression of NK cell surface receptors

a) Raw data of cytokines and chemokines levels quantified by ECLIA in the supernatants from NK cells that were cultured with IL-2 in presence or absence of IL-35 for 7 days and then activated by IL-12+IL-18 stimulations for 16h. Mean values ± S.D are shown (n = 6 individual donors). **B)** Raw data of NKp30, NKp44, NKp46, NKG2D, DNAM1 expression analyzed by flow cytometry in NK cells after 7d-culture in IL-2 with or without IL-35. Mean values ± S.D are shown (n=7 individual donors). **C)** Representative heat mapping of surface marker intensity for NKp30, NKp44, NKp46, DNAM1, NKG2D expression in NK cells after 7d-culture in IL-2 with or without IL-35, as indicated in Fig. 3a. **D)** Raw data of TIGIT, NKG2A, TIM3, PD-L1, CD96, LAG3 expression analyzed by flow cytometry in NK cells after 7d-culture in IL-2 with or without IL-35. Mean values ± S.D are shown (n= 6 or 7 individual donors). **e)** Representative heat mapping of surface marker intensity for TIGIT, NKG2A, TIM3, PD-L1, CD96, LAG3 expression in NK cells after 7d-culture in IL-2 with or without IL-35, as indicated in Fig. 3a. Source data are provided as a Source Data file.

Supplementary Fig. 4 | scRNA-seq reveals transcriptional regulation and mechanisms involved in NK cell subsets' response to IL-35

a) Heatmap of top 10 markers tested with the Wilcoxon rank sum test separating the healthy human NK cells into subsets. Cells are plotted in columns according to subset source. Genes are shown in rows and ranked by adjusted p values < 0.05. Gene expression is color-coded on a scale based on the z-score distribution, from −2 (purple) to 2 (yellow). **b-c)** Violin plots and UMAP showing UCell score distribution across Seurat clusters or in UMAP space for $CD56^{Dim}$ signature or $CD56^{Bright}$ signature (listed in Table 2). Signature score is color-coded in the UMAP on a scale based on the z-score distribution, from −0.5 (blue) to +0.5 (red). **d)** UMAP of AdaptNK (left), ConvNK (middle), and ProlifNK (right). Cells are colored according to the treatment received. **e)** Violin plots and UMAP showing UCell score distribution in presence or absence of IL-35 or in UMAP space for an ILC1-like signature (listed in Table 2) among

AdaptNK (left), ConvNK (middle) and ProlifNK (right). Signature score is color-coded in the UMAPs on a scale based on the z-score distribution, from −1 (blue) to +1 (red).

Supplementary Fig. 5 | IL-35 drives the conversion of human NK cells into an irreversible ILC1-like phenotype

a) Representative flow cytometry plots (left) and quantification of MFI surface residency markers' expression (right) after 8d-culture in IL-2 with or without IL-35, as indicated in Fig. 3a. Mean values ± S.D are shown (n=3 or 4 individual donors). **b)** Representative two-dimensional T-sne plots showing NK cell clustering based on surface residency markers' expression (CD9, CD103, CD49a) (n=3 to 4 individual donors). **c)** Representative flow cytometry plots for CD9 and CD103 expression in NK cells after 2, 4 and 8 days in culture with IL-2 or IL-2+IL-35. Colors represents CD49a intensity, from lowest expression in dark green to highest expression in red. **d)** Representative heat mapping of surface marker intensity for CD9, CD103, CD49a expression in NK cells after 8d-culture in IL-2 with or without IL-35. **e)** Schematic representation of the protocol used to assess reversibility of IL-35 effects on NK cell phenotype and cytokine production. Images were provided by Servier Medical Art (https://smart.servier.com/), licensed under CC BY 4.0 (https://creativecommons.org/licenses/by/4.0/)." Healthy donors' blood NK cells were cultured in low dose IL-2 (100 UI/mL) with or without IL-35 (100ng/ml) for 5 days and then were washed and cultured either in IL-2 or IL-2+IL-35 for 2 days. After a total period of 7 days, NK cells were activated with IL-12+IL-18 for 16h and analysed for cytokine production and phenotyping. Statistical significance was determined using paired T test. Source data are provided as a Source Data file.

Supplementary Fig. 6 | IL-35-triggered autocrine TGF-β drives NK cell dysfunction and conversion into ILC1-like cells in response to IL-35

a) Schematic representation of the protocol used to assess reversibility of TGF-β effects on NK cell phenotype and cytokine production. Images were provided by Servier Medical Art (https://smart.servier.com/), licensed under CC BY 4.0 (https://creativecommons.org/licenses/by/4.0/)." Healthy donors' blood NK cells were cultured in low dose IL-2 (100 UI/mL) with or without TGF-β (1ng/ml) for 5 days and then were washed and culture either in IL-2 or IL-2+ TGF-β for 2 days. After a total period of 7 days, NK cells were activated with IL-12+IL-18 for 16h and analysed for cytokine production and phenotyping. **b)** Quantification (%) of $CD9^+$ $CD103^+$ NK (right) after 5 days (5d) in culture with IL-2 with or without IL-35 and at day 7 following 48h (2d) in indicated culture conditions. Mean values ± S.D are shown (n= 3 to 4 individual donors). **c)** Quantification of IFN-γ expression in NK cells at day 7 following 48h (2d) in indicated culture conditions.

Results are expressed as relative MFI compared to control condition IL-2 (5d) -> IL-2 (2d). Mean values ± S.D are shown (n=4 individual donors). **d)** Supernatants from FACS-sorted NKG2C^{+} and NKG2C^{-} NK cells cultured for 24h in the presence of medium versus IL-12 and IL-18 with or without IL-35 were collected to quantify active TGF-β1 by ELISA Mean values ± S.D are shown (n=2 to 3 individual donors). **e)** Representative flow cytometry plots for CD9 and CD103 expression in NK cells after 8 days in culture with IL-2, IL-2+TGF-β, IL-2+TGF-β+TGF-βR inhibitor (Galunisertib) and IL-2+TGF-β+anti-TGF-β1/2/3 neutralizing antibody. **f)** Representative flow cytometry plots (left) and quantification of EOMES expression (right) after 8d-culture in IL-2 with or without IL-35 and TGF-βR inhibitor (Galunisertib). Results are expressed as relative MFI compared to control condition without IL-35. Mean values ± S.D are shown (n = 7 individual donors. Source data are provided as a Source Data file.

Supplementary Fig. 7 | NK/ILC1-like cells expressing IL-35R are present in tumors and IL-35 is associated with poor prognosis in cancer

a) UMAP embedding of an individual scRNA-seq dataset including both immune and non-immune cell populations, following quality control, standard preprocessing, and upstream analysis. **b)** UMAP embedding of the integrated pan-cancer single-cell RNA-seq atlas, generated by merging four individual datasets using the Harmony integration algorithm. **c)** Bar plot showing the cellular composition of each dataset, illustrating the relative proportions of immune and non-immune populations. **d)** Dot plot displaying average expression levels of IL-35 cytokine and IL-35 receptor subunits across selected immune cell populations. Dot size represents the percentage of cells expressing each gene within a given population, and color intensity reflects the scaled average expression, plotted on a uniform scale. **e)** Bubble maps showing the correlation coefficient for *EBI3* and *FOXP3* and for *IL12A* and *FOXP3* in TCGA datasets : adrenocortical carcinoma (ACC), bladder urothelial carcinoma (BLCA), breast invasive carcinoma (BRCA), cervical carcinoma (CESC), cholangiosarcoma (CHOL), colorectal adenocarcinoma (COAD), diffuse large B-cell lymphoma (DLBC), esophageal carcinoma (ESCA), glioblastoma multiforme (GBM), head and neck squamous cell carcinoma (HNSC), kidney chromophobe carcinoma (KICH), kidney clear renal cell carcinoma (KIRC), kidney papillary cell carcinoma (KIRP), lower grade glioma (LGG), liver hepatocellular carcinoma (LIHC), lung adenocarcinoma (LUAD), lung squamous cell carcinoma (LUSC), mesothelioma (MESO), ovarian serous cystadenocarcinoma (OV), pancreatic adenocarcinoma (PAAD), paraganglioma & pheochromocytoma (PCPG), prostate adenocarcinoma (PRAD), rectum adenocarcinoma (READ), sarcoma (SARC), skin cutaneous metastatic melanoma (SKCM), stomach adenocarcinoma (STAD), testicular germ cell cancer (TGCT), thyroid carcinoma (THCA), thymoma (THYM), uterine corpus endometrial carcinoma (UCEC), uterine carcinosarcoma (UCS) and uveal melanoma (UVM). Positive

(green) and negative (red) correlation are highlighted, based on p-val<0.05. Dots size represents absR, Pearson correlation coefficient.

Supplementary Fig. 8 | Gating strategies for flow cytometry analyses of NK cells

a) Gating strategy used to evaluate the purity of MACS-sorted NK cells from PBMCs by negative immune-selection using the Human NK cell isolation kit (Miltenyi). **b)** Gating strategy to FACS-sort NKG2C$^-$ (left) and NKG2C$^+$ (right) NK cells after MACS purification. **c,d)** Purity of NKG2C$^-$ **(c)** and NKG2C$^+$ **(d)** FACS-sorted NK cells.

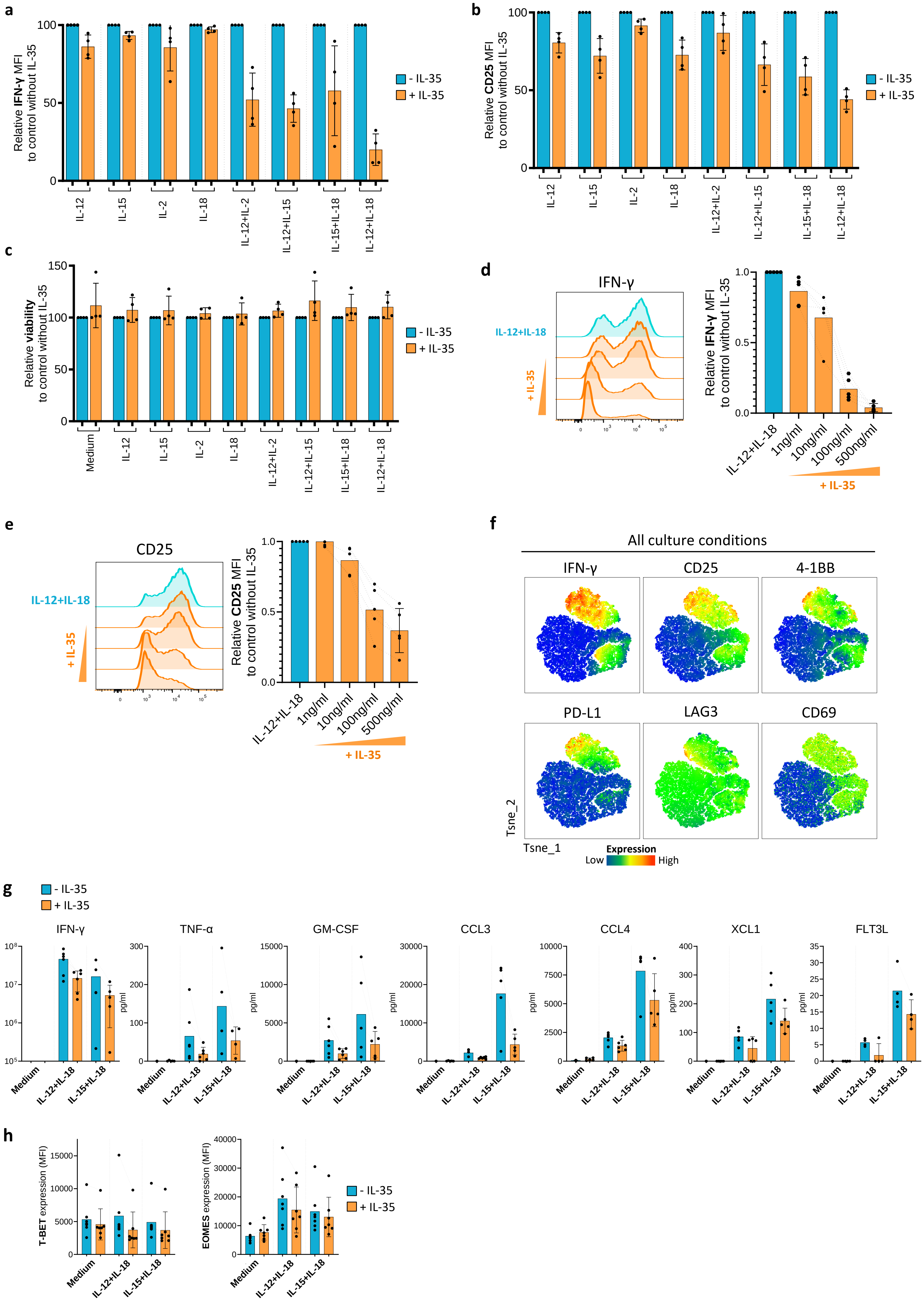

a
Relative IFN-γ MFI to control without IL-35
- IL-35
+ IL-35
IL-12
IL-15
IL-2
IL-18
IL-12+IL-2
IL-12+IL-15
IL-15+IL-18
IL-12+IL-18
b
Relative CD25 MFI to control without IL-35
c
Relative viability to control without IL-35
Medium
d
IFN-γ
IL-12+IL-18
+ IL-35
Relative IFN-γ MFI to control without IL-35
1ng/ml
10ng/ml
100ng/ml
500ng/ml
e
CD25
Relative CD25 MFI to control without IL-35
f
All culture conditions
IFN-γ
CD25
4-1BB
PD-L1
LAG3
CD69
Tsne_2
Tsne_1
Expression
Low
High
g
TNF-α
GM-CSF
CCL3
CCL4
XCL1
FLT3L
pg/ml (log10)
pg/ml
h
T-BET expression (MFI)
EOMES expression (MFI)

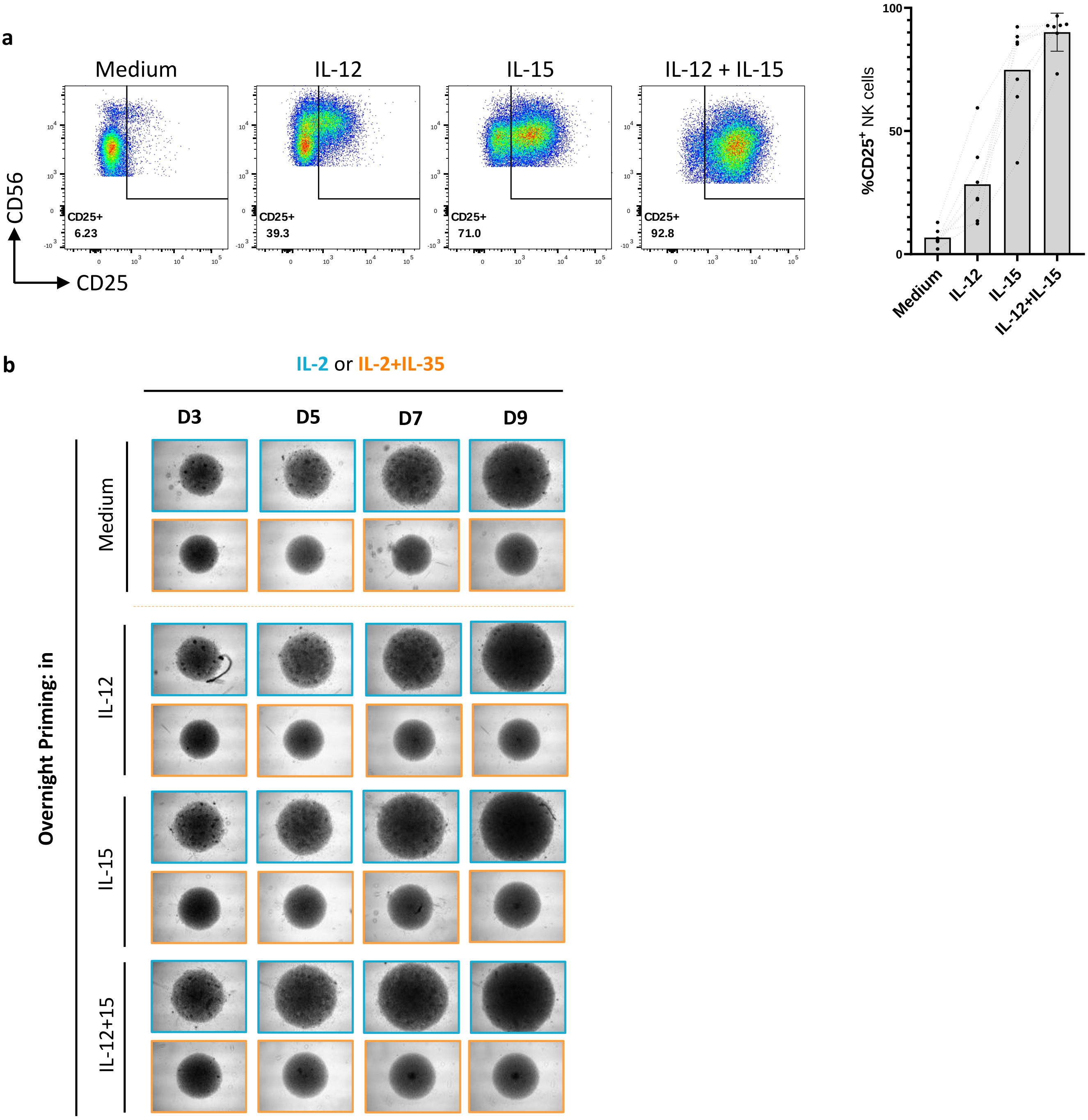
a
Medium
IL-12
IL-15
IL-12 + IL-15
CD56
CD25
CD25+
6.23
CD25+
39.3
CD25+
71.0
CD25+
92.8
%CD25+ NK cells
100
50
0
Medium
IL-12
IL-15
IL-12+IL-15
b
IL-2 or IL-2+IL-35
D3
D5
D7
D9
Overnight Priming: in
Medium
IL-12
IL-15
IL-12+15

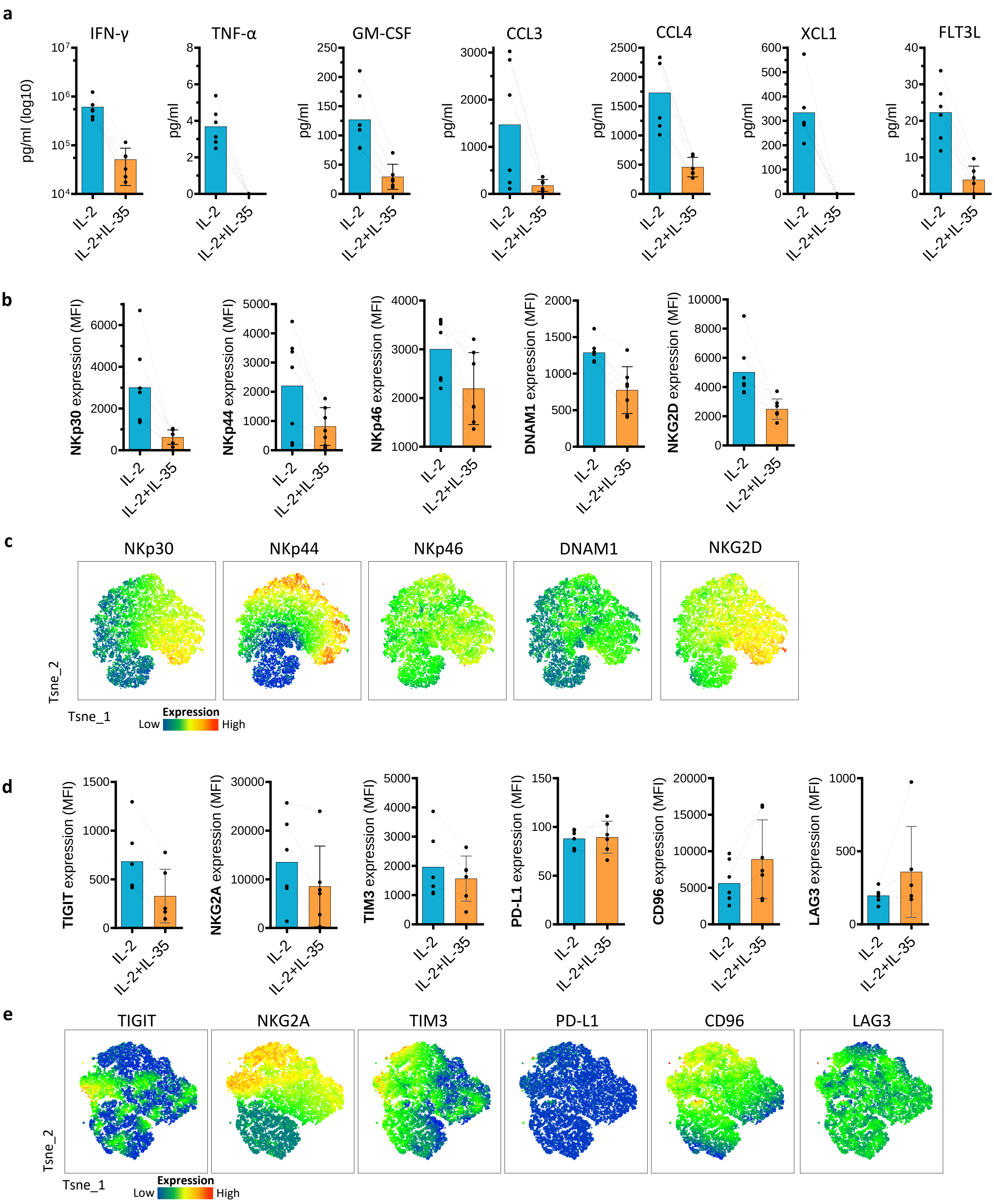

a
IFN-γ
TNF-α
GM-CSF
CCL3
CCL4
XCL1
FLT3L
pg/ml (log10)
pg/ml
IL-2
IL-2+IL-35
b
NKp30 expression (MFI)
NKp44 expression (MFI)
NKp46 expression (MFI)
DNAM1 expression (MFI)
NKG2D expression (MFI)
c
NKp30
NKp44
NKp46
DNAM1
NKG2D
Tsne_2
Tsne_1
Expression
Low
High
d
TIGIT expression (MFI)
NKG2A expression (MFI)
TIM3 expression (MFI)
PD-L1 expression (MFI)
CD96 expression (MFI)
LAG3 expression (MFI)
e
TIGIT
NKG2A
TIM3
PD-L1
CD96
LAG3

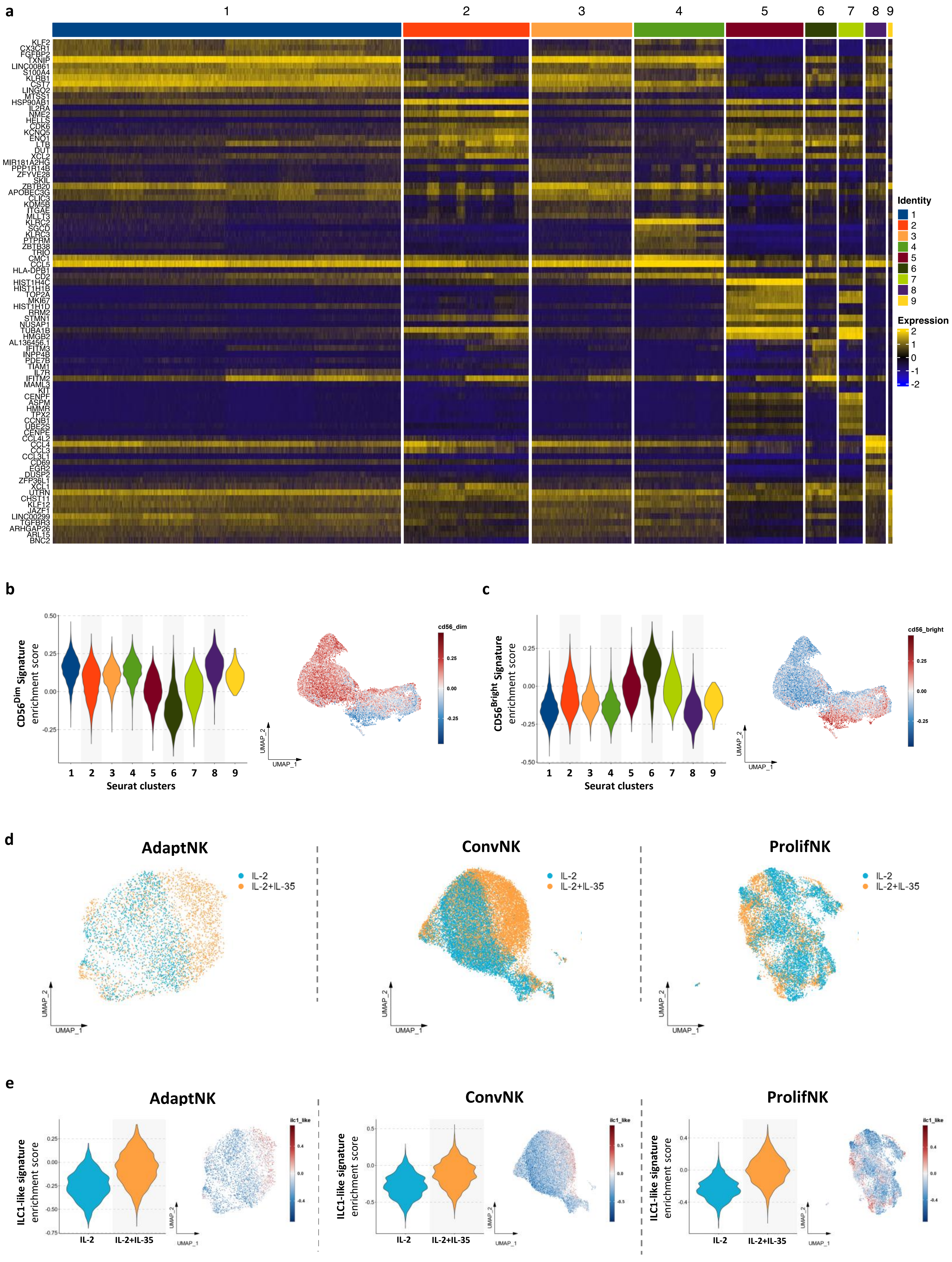

a
1
2
3
4
5
6
7
8
9
KLF2
CX3CR1
FGFBP2
TXNIP
LINC00861
S100A4
KLRB1
CST7
LINGO2
MTSS1
HSP90AB1
IL2RA
NME2
HELLS
CDK6
KCNQ5
ENO1
LTB
DUT
XCL2
MIR181A2HG
PPP1R14B
ZFYVE28
SKIL
ZBTB20
APOBEC3G
CLIC3
KDM5B
ITGAE
MLLT3
KLRC2
SGCD
KLRC3
PTPRM
ZBTB38
TRIO
CMC1
CCL5
HLA-DPB1
CD2
HIST1H4C
HIST1H1B
TOP2A
MKI67
HIST1H1D
RRM2
STMN1
NUSAP1
TUBA1B
HMGB2
AL136456.1
IFITM3
INPP4B
PDE7B
TIAM1
IL7R
IFITM2
MAML3
KIT
CENPF
ASPM
HMMR
TPX2
CCNB1
UBE2S
CENPE
CCL4L2
CCL4
CCL3
CCL3L1
CD69
EGR2
DUSP2
ZFP36L1
XCL1
UTRN
CHST11
KLF12
JAZF1
LINC00299
TGFBR3
ARHGAP26
ARL15
BNC2
Identity
1
2
3
4
5
6
7
8
9
Expression
2
1
0
-1
-2
b
CD56^Dim Signature enrichment score
0.50
0.25
0.00
-0.25
1 2 3 4 5 6 7 8 9
Seurat clusters
cd56_dim
0.25
0.00
-0.25
UMAP_2
UMAP_1
c
CD56^Bright Signature enrichment score
0.25
0.00
-0.25
-0.50
1 2 3 4 5 6 7 8 9
Seurat clusters
cd56_bright
0.25
0.00
-0.25
UMAP_2
UMAP_1
d
AdaptNK
IL-2
IL-2+IL-35
UMAP_2
UMAP_1
ConvNK
IL-2
IL-2+IL-35
UMAP_2
UMAP_1
ProlifNK
IL-2
IL-2+IL-35
UMAP_2
UMAP_1
e
AdaptNK
ILC1-like signature enrichment score
0.25
0.00
-0.25
-0.50
-0.75
IL-2
IL-2+IL-35
ilc1_like
0.4
0.0
-0.4
UMAP_2
UMAP_1
ConvNK
ILC1-like signature enrichment score
0.5
0.0
-0.5
IL-2
IL-2+IL-35
ilc1_like
0.5
0.0
-0.5
UMAP_2
UMAP_1
ProlifNK
ILC1-like signature enrichment score
0.4
0.0
-0.4
IL-2
IL-2+IL-35
ilc1_like
0.4
0.0
-0.4
UMAP_2
UMAP_1

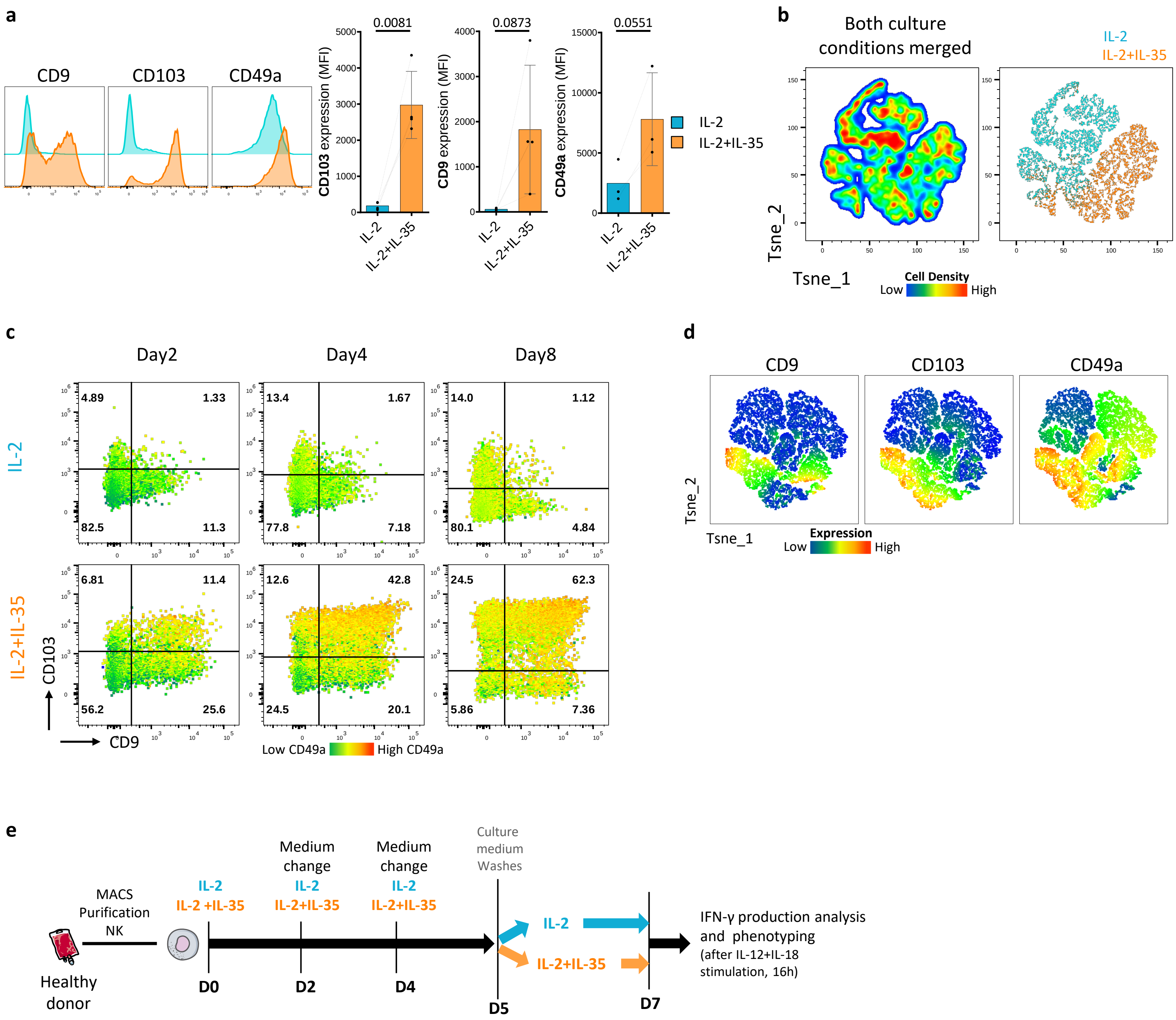
a
CD9
CD103
CD49a
0.0081
0.0873
0.0551
CD103 expression (MFI)
CD9 expression (MFI)
CD49a expression (MFI)
IL-2
IL-2+IL-35
b
Both culture conditions merged
IL-2
IL-2+IL-35
Tsne_2
Tsne_1
Cell Density
Low
High
c
Day2
Day4
Day8
IL-2
IL-2+IL-35
CD103
CD9
Low CD49a
High CD49a
d
CD9
CD103
CD49a
Tsne_2
Tsne_1
Expression
Low
High
e
MACS
Purification
NK
Healthy
donor
D0
D2
D4
D5
D7
Medium
change
Culture
medium
Washes
IL-2
IL-2 +IL-35
IL-2+IL-35
IFN-γ production analysis
and phenotyping
(after IL-12+IL-18
stimulation, 16h)

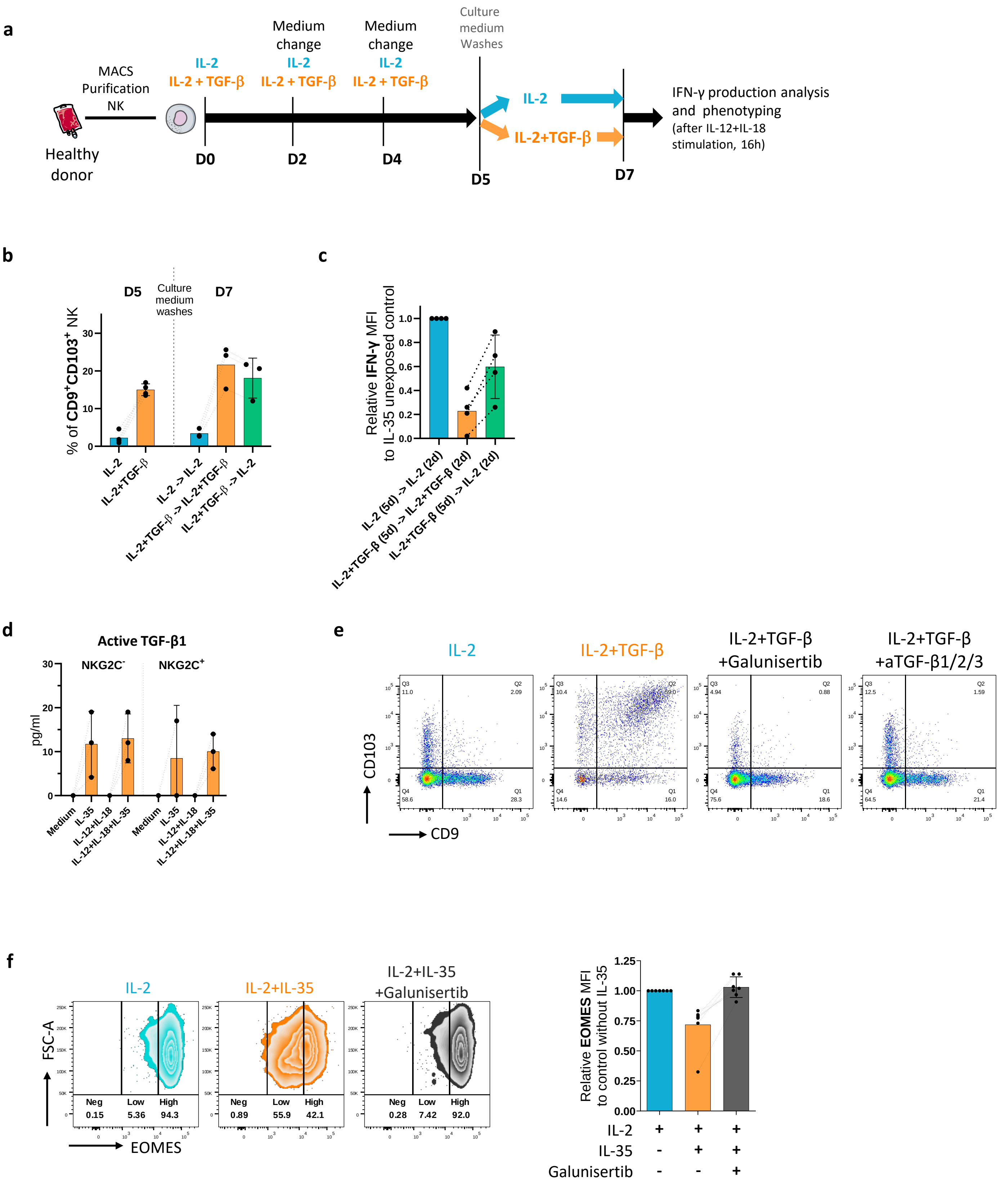

a
Healthy donor
MACS Purification NK
D0
D2
D4
D5
D7
IL-2
IL-2 + TGF-β
Medium change
Culture medium Washes
IL-2+TGF-β
IFN-γ production analysis and phenotyping
(after IL-12+IL-18 stimulation, 16h)
b
D5
D7
Culture medium washes
% of CD9+CD103+ NK
IL-2
IL-2+TGF-β
IL-2 -> IL-2
IL-2+TGF-β -> IL-2+TGF-β
IL-2+TGF-β -> IL-2
c
Relative IFN-γ MFI to IL-35 unexposed control
IL-2 (5d) -> IL-2 (2d)
IL-2+TGF-β (5d) -> IL-2+TGF-β (2d)
IL-2+TGF-β (5d) -> IL-2 (2d)
d
Active TGF-β1
NKG2C-
NKG2C+
pg/ml
Medium
IL-35
IL-12+IL-18
IL-12+IL-18+IL-35
e
IL-2
IL-2+TGF-β
IL-2+TGF-β +Galunisertib
IL-2+TGF-β +aTGF-β1/2/3
CD103
CD9
f
IL-2
IL-2+IL-35
IL-2+IL-35 +Galunisertib
FSC-A
EOMES
Neg
Low
High
Relative EOMES MFI to control without IL-35
IL-2
IL-35
Galunisertib

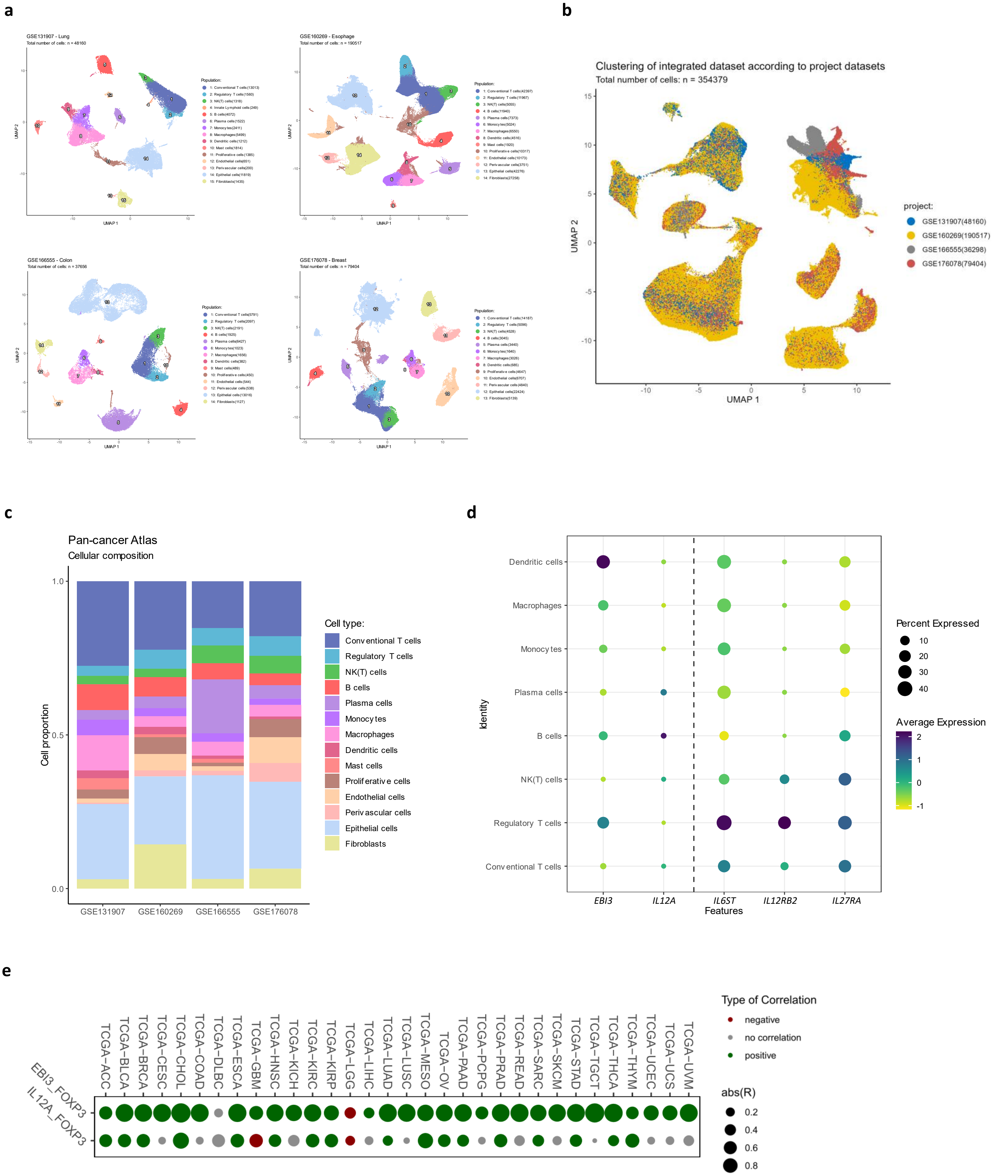
a
GSE131907 - Lung
Total number of cells: n = 48160
GSE160269 - Esophage
Total number of cells: n = 190517
GSE166555 - Colon
Total number of cells: n = 37656
GSE176078 - Breast
Total number of cells: n = 79404
b
Clustering of integrated dataset according to project datasets
Total number of cells: n = 354379
UMAP 1
UMAP 2
project:
GSE131907(48160)
GSE160269(190517)
GSE166555(36298)
GSE176078(79404)
c
Pan-cancer Atlas
Cellular composition
Cell proportion
Cell type:
Conventional T cells
Regulatory T cells
NK(T) cells
B cells
Plasma cells
Monocytes
Macrophages
Dendritic cells
Mast cells
Proliferative cells
Endothelial cells
Perivascular cells
Epithelial cells
Fibroblasts
GSE131907
GSE160269
GSE166555
GSE176078
d
Identity
Dendritic cells
Macrophages
Monocytes
Plasma cells
B cells
NK(T) cells
Regulatory T cells
Conventional T cells
EBI3
IL12A
IL6ST
IL12RB2
IL27RA
Features
Percent Expressed
Average Expression
e
EBI3_FOXP3
IL12A_FOXP3
TCGA-ACC
TCGA-BLCA
TCGA-BRCA
TCGA-CESC
TCGA-CHOL
TCGA-COAD
TCGA-DLBC
TCGA-ESCA
TCGA-GBM
TCGA-HNSC
TCGA-KICH
TCGA-KIRC
TCGA-KIRP
TCGA-LGG
TCGA-LIHC
TCGA-LUAD
TCGA-LUSC
TCGA-MESO
TCGA-OV
TCGA-PAAD
TCGA-PCPG
TCGA-PRAD
TCGA-READ
TCGA-SARC
TCGA-SKCM
TCGA-STAD
TCGA-TGCT
TCGA-THCA
TCGA-THYM
TCGA-UCEC
TCGA-UCS
TCGA-UVM
Type of Correlation
negative
no correlation
positive
abs(R)

a

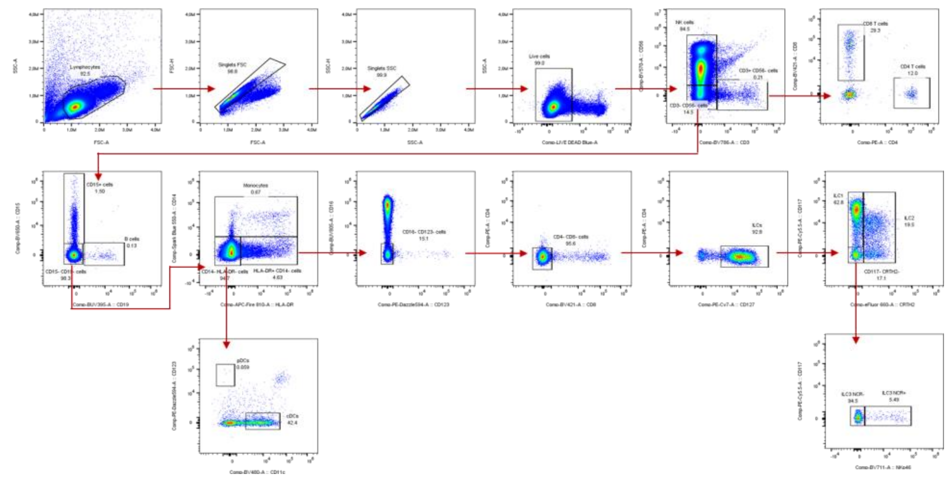

b

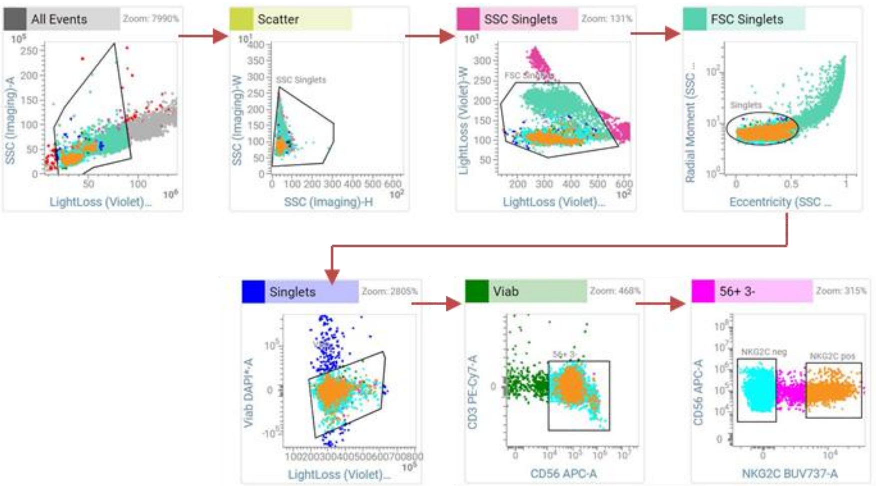

Population	Events	% Parent	% Total
All Events	20,000	N/A	100.00 %
Saturated	556	2.78 %	2.78 %
Unsaturated	19,444	97.22 %	97.22 %
Scatter	18,163	90.82 %	90.82 %
SSC Singlets	18,161	99.99 %	90.81 %
FSC Singlets	17,408	95.85 %	87.04 %
Singlets	12,812	73.60 %	64.06 %
Viab	12,611	98.43 %	63.06 %
56+ 3-	12,236	97.03 %	61.18 %
NKG2C neg	9,290	75.92 %	46.45 %
NKG2C pos	2,306	18.85 %	11.53 %

c

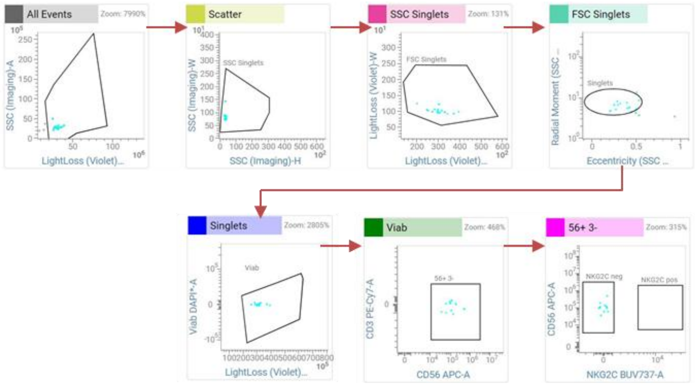

Population	Events	% Parent	% Total
All Events	19	N/A	100.00 %
Saturated	0	0.00 %	0.00 %
Unsaturated	19	100.00 %	100.00 %
Scatter	16	84.21 %	84.21 %
SSC Singlets	16	100.00 %	84.21 %
FSC Singlets	16	100.00 %	84.21 %
Singlets	11	68.75 %	57.89 %
Viab	11	100.00 %	57.89 %
56+ 3-	11	100.00 %	57.89 %
NKG2C neg	11	100.00 %	57.89 %
NKG2C pos	0	0.00 %	0.00 %

d

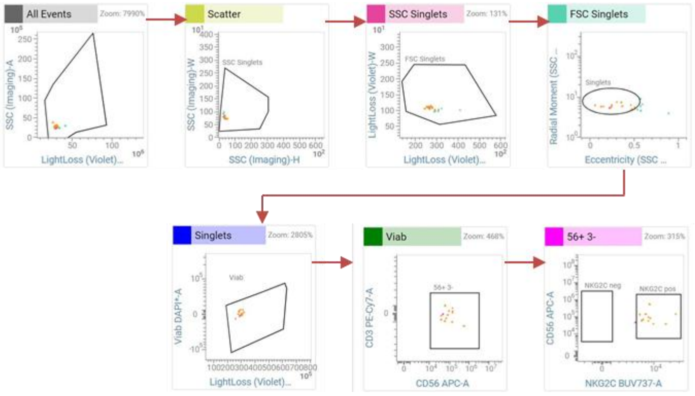

Population	Events	% Parent	% Total
All Events	20	N/A	100.00 %
Saturated	0	0.00 %	0.00 %
Unsaturated	20	100.00 %	100.00 %
Scatter	20	100.00 %	100.00 %
SSC Singlets	20	100.00 %	100.00 %
FSC Singlets	20	100.00 %	100.00 %
Singlets	13	65.00 %	65.00 %
Viab	13	100.00 %	65.00 %
56+ 3-	13	100.00 %	65.00 %
NKG2C neg	0	0.00 %	0.00 %
NKG2C pos	12	92.31 %	60.00 %
