# Supplementary material for: Interleukin-35 impairs human NK cell effector functions and induces their ILC1-like conversion with tissue residency features
Source: Nat Commun. 2025 Jul 3;16:6135. doi: 10.1038/s41467-025-61196-0 (PMC12229632; doi:10.1038/s41467-025-61196-0)
Supplement: Supplementary file 2 — Reporting Summary [file 41467_2025_61196_MOESM2_ESM.pdf]

nature portfolio

Corresponding author(s): Nathalie BENDRISS-VERMARE

Last updated by author(s): May 28, 2025

Reporting Summary

Nature Portfolio wishes to improve the reproducibility of the work that we publish. This form provides structure for consistency and transparency in reporting. For further information on Nature Portfolio policies, see our Editorial Policies and the Editorial Policy Checklist.

Statistics

For all statistical analyses, confirm that the following items are present in the figure legend, table legend, main text, or Methods section.

n/a	Confirmed	
☐	☒	The exact sample size (*n*) for each experimental group/condition, given as a discrete number and unit of measurement
☐	☒	A statement on whether measurements were taken from distinct samples or whether the same sample was measured repeatedly
☐	☒	The statistical test(s) used AND whether they are one- or two-sided<br>*Only common tests should be described solely by name; describe more complex techniques in the Methods section.*
☒	☐	A description of all covariates tested
☐	☒	A description of any assumptions or corrections, such as tests of normality and adjustment for multiple comparisons
☐	☒	A full description of the statistical parameters including central tendency (e.g. means) or other basic estimates (e.g. regression coefficient) AND variation (e.g. standard deviation) or associated estimates of uncertainty (e.g. confidence intervals)
☐	☒	For null hypothesis testing, the test statistic (e.g. *F*, *t*, *r*) with confidence intervals, effect sizes, degrees of freedom and *P* value noted<br>*Give P values as exact values whenever suitable.*
☒	☐	For Bayesian analysis, information on the choice of priors and Markov chain Monte Carlo settings
☒	☐	For hierarchical and complex designs, identification of the appropriate level for tests and full reporting of outcomes
☐	☒	Estimates of effect sizes (e.g. Cohen's *d*, Pearson's *r*), indicating how they were calculated

Our web collection on statistics for biologists contains articles on many of the points above.

Software and code

Policy information about availability of computer code

Data collection: All experimental data were collected and generated as detailed in the Material&Methods section.

Data analysis: For flowcytometry analysis : FlowJo 10.6.2 Software (Tree Star).

All codes used for analysis in this study were based on the following R packages and Github link :
-For single cell analysis:
CellRanger v7.1.0
Seurat (v5.1.0) - https://satijalab.org/seurat/
SCP (v0.5.6) https://github.com/hermespara/SCP_HP
clusterProfiler (v4.2.2) https://github.com/YuLab-SMU/clus
terProfiler
UCell (v2.10.1) https://github.com/carmonalab/UCell
harmony (v1.2.3) https://github.com/immunogenomics/harmony
effsize (v0.8.0) https://github.com/mtorchiano/effsize
msigdbr (v7.5.1) https://github.com/igordot/msigdbr
MAST (v1.20.0) https://github.com/RGLab/MAST/

-Data visualization and manipulation:
Dplyr (v1.1.04) https://github.com/tidyverse/dplyr

ggsci (v2.9) https://github.com/nanxstats/ggsci
ggpubr (v0.4.0) https://github.com/kassambara/ggpubr
ggeasy (v0.1.3) https://github.com/jonocarroll/ggeasy
tibble (v3.2.1) https://github.com/tidyverse/tibble
DT (v0.26) https://github.com/rstudio/DT
kableExtra (v1.3.4) https://github.com/haozhu233/kableExtra
knitr (v1.42) https://github.com/yihui/knitr
Nebulosa (v1.16.0) https://github.com/powellgenomicslab/Nebulosa
scCustomize (v3.0.1) https://github.com/samuel-marsh/scCustomize
RColorBrewer (v1.1-3) https://github.com/cran/RColorBrewer
EnhancedVolcano (v1.12.0) https://github.com/kevinblighe/EnhancedVolcano
SCpubr (v1.1.2.9000) https://github.com/enblacar/SCpubr
stringr (v1.5.0) https://github.com/tidyverse/stringr
tidyr (v1.3.0) https://tidyr.tidyverse.org/
dittoSeq (v1.18.0) https://www.bioconductor.org/packages/release/bioc/html/dittoSeq.html

-For RNAseq analysis (TCGA) :
R software v 4.4.2
survival (v3.7-0) https://github.com/therneau/survival
survminer (v0.5.0) https://rpkgs.datanovia.com/survminer/index.html
ggplot2 (v3.5.1) https://ggplot2.tidyverse.org/

All bioinformatic tools and methods used in this manuscript have been published previously and no custom code was used.
For further information please contact the corresponding author.
Source data are provided with this paper.

For manuscripts utilizing custom algorithms or software that are central to the research but not yet described in published literature, software must be made available to editors and reviewers. We strongly encourage code deposition in a community repository (e.g. GitHub). See the Nature Portfolio guidelines for submitting code & software for further information.

Data

Policy information about availability of data

All manuscripts must include a data availability statement. This statement should provide the following information, where applicable:

- Accession codes, unique identifiers, or web links for publicly available datasets
- A description of any restrictions on data availability
- For clinical datasets or third party data, please ensure that the statement adheres to our policy

The data that support the findings of this study are available from the corresponding author on request and the scRNA sequencing data that support some findings of this study have been deposited in Gene Expression Omnibus – NCBI (GEO) with the GEO ID GSE256137. Source data are provided with this paper

Research involving human participants, their data, or biological material

Policy information about studies with human participants or human data. See also policy information about sex, gender (identity/presentation), and sexual orientation and race, ethnicity and racism.

Reporting on sex and gender	Our research findings do not apply to only one sex or gender. Our study involves human blood from EFS (France) and information on sex and/or gender was not collected and not considered in the study design.
Reporting on race, ethnicity, or other socially relevant groupings	Our study does not use the concepts of race and/or ethnicity
Population characteristics	Healthy donor blood was obtained from EFS (France) and population characteristics were not considered
Recruitment	not applicable
Ethics oversight	not applicable

Note that full information on the approval of the study protocol must also be provided in the manuscript.

Field-specific reporting

Please select the one below that is the best fit for your research. If you are not sure, read the appropriate sections before making your selection.

☒ Life sciences ☐ Behavioural & social sciences ☐ Ecological, evolutionary & environmental sciences

For a reference copy of the document with all sections, see nature.com/documents/nr-reporting-summary-flat.pdf

Life sciences study design

All studies must disclose on these points even when the disclosure is negative.

Sample size	The sample size calculation was performed with the online software (Experimental design assistant https://eda.nc3rs.org.uk/eda/login/auth) according to the estimated effect size and standard deviation (Variability base in previous published results when possible) and adjusted if necessary to tissue availability .
Data exclusions	no data were excluded from the analyses
Replication	All the experiments were replicated at least two times => 3 independent experiments at least for each figure
Randomization	This is not relevant to our study
Blinding	Blinding is not relevant to our study (no clinical trial)

Reporting for specific materials, systems and methods

We require information from authors about some types of materials, experimental systems and methods used in many studies. Here, indicate whether each material, system or method listed is relevant to your study. If you are not sure if a list item applies to your research, read the appropriate section before selecting a response.

Materials & experimental systems

n/a	Involved in the study	
☐	☒	Antibodies
☐	☒	Eukaryotic cell lines
☒	☐	Palaeontology and archaeology
☒	☐	Animals and other organisms
☒	☐	Clinical data
☒	☐	Dual use research of concern
☒	☐	Plants

Methods

n/a	Involved in the study	
☒	☐	ChIP-seq
☐	☒	Flow cytometry
☒	☐	MRI-based neuroimaging

Antibodies

Antibodies used	mAbs used in our study are provided in the Methods section and supplemental table 1
Validation	Blood for FCM as available on the manufacturer's websites.

Eukaryotic cell lines

Policy information about cell lines and Sex and Gender in Research

Cell line source(s)	K562 cell line was provided from ATCC
Authentication	No authentification needed as K562 was provided from ATCC
Mycoplasma contamination	K562 cell line was tested negative for mycoplasma contamination
Commonly misidentified lines (See ICLAC register)	not applicable

Plants

Seed stocks	not applicable
Novel plant genotypes	not applicable
Authentication	not applicable

Flow Cytometry

Plots

Confirm that:

- [x] The axis labels state the marker and fluorochrome used (e.g. CD4-FITC).
- [x] The axis scales are clearly visible. Include numbers along axes only for bottom left plot of group (a 'group' is an analysis of identical markers).
- [x] All plots are contour plots with outliers or pseudocolor plots.
- [x] A numerical value for number of cells or percentage (with statistics) is provided.

Methodology

Sample preparation	Each sample preparation is detailed in the Methods Section
Instrument	Cells were analyzed on a LSR-Fortessa 4 lasers (BD Biosciences) or spectral flow cytometer AURORA (Cytek).
Software	Data were processed using the FlowJo 10.6.2 Software (Tree Star).
Cell population abundance	Total NK cells were purified from PBMCs by negative immune-selection using the Human NK cell isolation kit (Miltenyi) following the manufacturer's instructions. To evaluate the purity, MACS-sorted cells were stained with CD3, CD56, and DAPI and the purity always exceeded 90%. For experiments distinguishing NKG2C- and NKG2C+ NK cells, cells were purified with the same isolation kit and further stained with antibodies for CD56, CD3 and NKG2C, FACS sorted using a FACSDiscover™ S8 Cell Sorter (BD). Cell viability was determined by DAPI staining (1 µg/mL, D1306 Invitrogen) NKG2C- and NKG2C+ cells were collected in cRPMI and purity always exceeded 98%.
Gating strategy	For all experiments, cells were gated on morphology (FSC-A/SSC-A), then doublets were excluded (FSC-A/FSC-H), then among viable cells CD3- CD14- CD19- CD56+ cells were considered. When necessary, an additional gate distinguishing NKG2C- and NKG2C+ cells was added.

- [x] Tick this box to confirm that a figure exemplifying the gating strategy is provided in the Supplementary Information.
